# Investigating Size-Dependent Conductive Properties on Individual Si Nanowires

**DOI:** 10.1186/s11671-020-3277-3

**Published:** 2020-03-02

**Authors:** X. F. Hu, S. J. Li, J. Wang, Z. M. Jiang, X. J. Yang

**Affiliations:** State Key Laboratory of Surface Physics, Fudan University, Shanghai, 200433 China

**Keywords:** Si nanowires, Conductive atomic force microscopy, Conductive property, Size dependence, Schottky barrier height

## Abstract

Periodically ordered arrays of vertically aligned Si nanowires (Si NWs) are successfully fabricated by nanosphere lithography combined with metal-assisted chemical etching. By adjusting the etching time, both the nanowires’ diameter and length can be well controlled. The conductive properties of such Si NWs and particularly their size dependence are investigated by conductive atomic force microscopy (CAFM) on individual nanowires. The results indicate that the conductance of Si NWs is greatly relevant to their diameter and length. Si NWs with smaller diameters and shorter lengths exhibit better conductive properties. Together with the I–V curve characterization, a possible mechanism is supposed with the viewpoint of size-dependent Schottky barrier height, which is further verified by the electrostatic force microscopy (EFM) measurements. This study also suggests that CAFM can act as an effective means to explore the size (or other parameters) dependence of conductive properties on individual nanostructures, which should be essential for both fabrication optimization and potential applications of nanostructures.

## Introduction

Silicon nanowires (Si NWs) have gained promising applications in electronic, photonic, optoelectronic and many other fields due to their high aspect ratio and unique electrical, thermoelectric and photoelectrical properties, as well as the compatibility with traditional silicon technology [1–5]. In the past decades, the researches of Si NWs have mainly focused on the growth improvements and property measurements. Many methods have been developed to prepare Si NWs, including bottom-up methods such as vapor-liquid-solid method, chemical vapor deposition, and molecular beam epitaxy [6–10] and top-down approaches using electron-beam lithography, reactive ion etching or metal-assisted chemical etching [11–16]. Among these methods, nanosphere lithography (NSL) combined with metal-assisted chemical etching (MACE) has been intensively adopted to fabricate large-area ordered arrays of vertically aligned Si NWs for its simplicity, low cost, and versatility [15–23]. The Si NWs achieved by MACE usually have very rough and even porous surfaces [18–21], which just makes them have a large specific surface area and excellent properties, leading to great application potentials in biosensors, thermoelectric devices, lithium-ion batteries, solar cells, etc. [22–24]. To realize those applications, it is essential to get a good understanding of their electrical properties. Nowadays two kinds of methods have often been applied to investigate the electrical properties of nanowires. One is carried out by ordinary macroscopic methods, which is relatively easy to do but can only provide averaged results over a large assembly of nanowires [24, 25]. The other is performed on individual nanowires with special fabricated single nanowire devices [21, 26–28], which could exclude the influence of size inhomogeneity but is not easy to achieve, especially when measuring the property dependence. Therefore, more convenient methods to study the electrical properties on individual NWs without complex nanofabrication are strongly required.

In recent decades, scanning probe microscopy (SPM)-based electrical measurements reveal themselves as powerful techniques for electrical characterizations at nanoscale [29, 30]. Among these SPM techniques, conductive atomic force microscopy (CAFM) has been successfully applied to study the conductive properties on single or individual nanostructures [30–32]. CAFM investigations on a variety of nanowires, such as ZnO, InAs, CdS, CdSe, GaAs, InAsSb, and Si NWs, have already been reported [33–38]. While most researches focused on the conductive properties of nanowires with fixed parameters, some investigations were carried out to explore the doping dependence of conductive properties [37–39]. The size dependencies of conductive properties of nanowires have been much less investigated yet. Only a few studies have been reported about the diameter-dependent conductive properties of nanowires, even not restricted to CAFM, and a considerable part of them dealt with the diameter dependence of nanowires’ resistivity [25, 40–42]. For example, a couple of researches on GaN nanowires found that the resistivity was high at a critical diameter (20 ~ 80 nm, dependent on fabrication methods) and kept unchanged beyond it [41, 43], whereas other researches on Si nanowires reported that the nanowire resistivity decreased with increased diameter in the range of tens to hundreds of nanometers [40, 44]. For semiconductor nanowires with metal contacts, Schottky barrier may play an important role in their conductive properties. Therefore, except for the resistivity, the Schottky barrier should be taken into consideration in the presence of semiconductor nanowires. Several papers have focused on the diameter dependence of Schottky barrier height (SBH), but the results are not consistent. For instance, Calahorra et al. calculated the SBH by solving Poisson’s equation in nanowire, and found a clear trend of increased barrier height with size reduction [45]. Similar diameter dependence was experimentally reported by Soudi et al. when investigating the diameter-dependent surface photovoltage and surface state density on ZnO single nanowire device. They found that the surface barrier height increased with the decreasing diameter (20–60 nm), which was interpreted by surface state density simulated using Poisson’s equation [46]. By contrast, scanning photocurrent microscopy measurements on single Si nanowire field-effect transistors by Yoon et al. revealed a contrary dependence, that is, the barrier height decreased with the decreased diameter due to the contribution of interface states [47]. Another experimental work by Mao et al. on single Pt/ZnO nanoneedle Schottky diodes also reported the barrier height decreased with the decreasing diameter, which was explained by a joule heating effect and/or electronic inhomogeneity of interface [48]. Therefore up to now, the diameter dependence of nanowires’ conductive properties has not reached a consensus yet and is far from being thoroughly understood. Especially, the size-dependent conductive properties as well as size-dependent SBH studies have not been reported on individual Si NWs fabricated by the MACE method, which have quite a rough surface for promising applications.

In this paper, periodic arrays of vertically aligned Si NWs with different diameters and lengths are prepared by the method of NSL combined with MACE. Both the diameter and length of SiNWs can be well controlled by adjusting the etching time. The conductive properties of individual Si NWs inside the array are investigated by CAFM without any further nanofabrication, which can study the size-dependent conductive properties on individual Si NWs conveniently by simply replacing the sample. The results demonstrate that the current measured on individual Si NWs is strongly dependent on the NWs’ diameter and length. Si NWs with smaller diameters and shorter lengths exhibit better conductive properties. From I–V curve fitting, size-dependent Schottky barrier heights can be obtained, which is found to act as a key factor to determine the size-dependent conductive properties of nanowires. Furthermore, similar size-dependent SBH was obtained by electrostatic force microscopy (EFM) measurements. Therefore, our study not only reveals the size-dependent properties of Si NWs but also suggests that CAFM can act as an effective means to explore the size (or other parameters) dependence of conductive properties on individual nanostructures.

## Materials and Methods

### Materials

The Si wafers were purchased from MTI (China). The suspensions (2.5 wt% in water) of polystyrene spheres (PS, 490 nm in diameter) were purchased from Duke Scientific (USA). Acetone, methanol, sulfuric acid, hydrogen peroxide, and hydrofluoric acid for fabricating Si NWs were purchased from Sinopharm Chemical Reagent (China). Deionized water (DI, 18.2 MΩ·cm) was obtained from an ultrafiltration system (Milli-Q, Millipore, Marlborough, MA).

### Fabrication of Si NWs

**Fig. 1:**
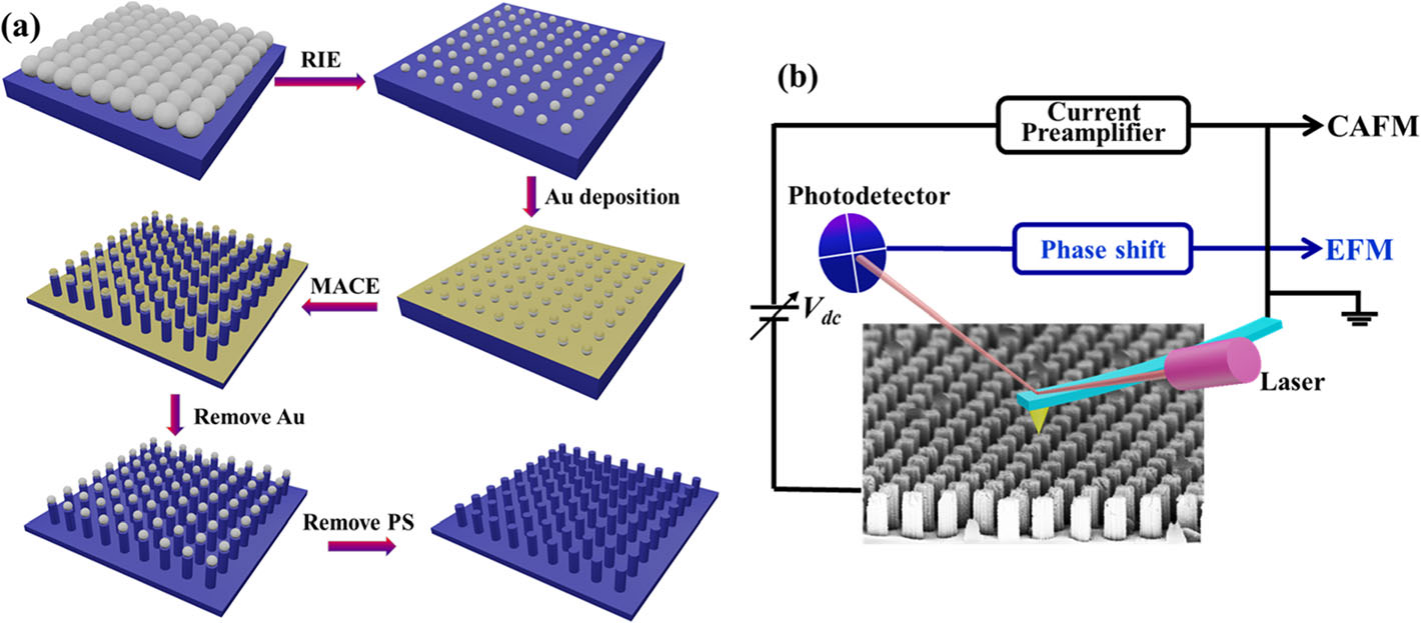
**a** Schematic illustration of the procedures to fabricate vertically aligned Si nanowire array. **b** Schematic diagram of the experimental set-ups for CAFM and EFM measurements on individual Si nanowires

Vertically ordered silicon nanowire arrays are fabricated by NSL combined with MACE, which have been described in detail in previous literatures [49, 50]. The major fabrication process is scheduled in Fig. 1a. Firstly, a monolayer of polystyrene spheres (PS) with a diameter of 490 nm was self-assembled onto the chemically cleaned planar Si wafer (n-type, 0.01 Ω cm) to form a hexagonal close-packed monolayer. Subsequently, the PS covered sample was etched by reactive ion etching (RIE, Trion Technology) (50 W, 70 mTorr) with O_2_ gas (20 sccm) to reduce the diameter of PS, which acted as the mask layer in the following procedures. Afterwards, a 20 nm Au film was deposited by ion sputtering onto the sample masked by the PS layer acted as a catalyst for the following MACE treatment. The sample was treated by MACE in HF (40%) and H_2_O_2_ (30%) mixed solution (volume ratio 4:1) at room temperature. In the MACE process, the Si surface covered with Au was effectively etched while that covered by PS (without Au) was protected, resulting in the formation of Si NWs. Finally, the remained Au layer and PS spheres were removed by soaking the sample in KI/I_2_ and tetrahydrofuran solutions, respectively. With this fabrication method, large-area periodic arrays of vertically aligned Si NW arrays can be obtained.

### Characterization of Si NWs

The morphology of fabricated Si NWs was checked by scanning electron microscopy (SEM, SIGMA300) while their structural properties were investigated by Raman spectroscopy (Jobin Yvon HR-Evolution 2 system) with an excitation wavelength of 532 nm and a low power of about 1 mW.

The electrical properties of Si NWs were investigated by CAFM and EFM with a commercial SPM equipment (Multimode V, Bruker Nano Surfaces), as diagrammed in Fig. 1b. Cr/Pt-coated tips (Multi75E-G, Budget Sensors, radius approximately 25 nm) were used for both CAFM and EFM measurements. In CAFM, the conductive tip was scanned over the sample surface in contact mode with a DC bias voltage applied to the substrate while the tip was grounded, and the current between the tip and sample was measured. As the surface anodic oxidation is serious under the positive sample biases, all the current images were measured at negative sample biases. Various negative voltages ranged from − 0.5 to − 3.0 V were tested in the CAFM experiments. It was found, when the bias voltage was set below − 1.5 V, the current was too small to be detected for samples with poor conductance. While the bias voltage was set as − 2.0 V or larger, the measurements were unstable, probably due to the damage of tip and/or sample under a large electrostatic field. Therefore, the bias voltage of − 1.5 V was chosen for current image measurements. The EFM measurements were carried out on Si NWs in the two-pass mode. In the first pass, it worked in tapping mode to get the topography image, while in the second pass the tip was lifted high enough to ignore Van der Waals force. In the lifted pass, a DC voltage was added between sample and grounded tip, and the phase shift signal induced by electrostatic force was detected. All the experiments were operated in a flowing N_2_ ambient for stable electrical measurements and the samples were pre-dipped in the HF solution (5%) for 30 s to effectively reduce the influence of the oxide layer on the conductive characterization. Since the measurements were carried out immediately after HF dipping, the reformed oxygen layer should be thin enough to be penetrated by the conductive tip and its effect on the conductance is minimal.

## Results and Discussions

### Fabrication of Si NWs

**Fig. 2:**
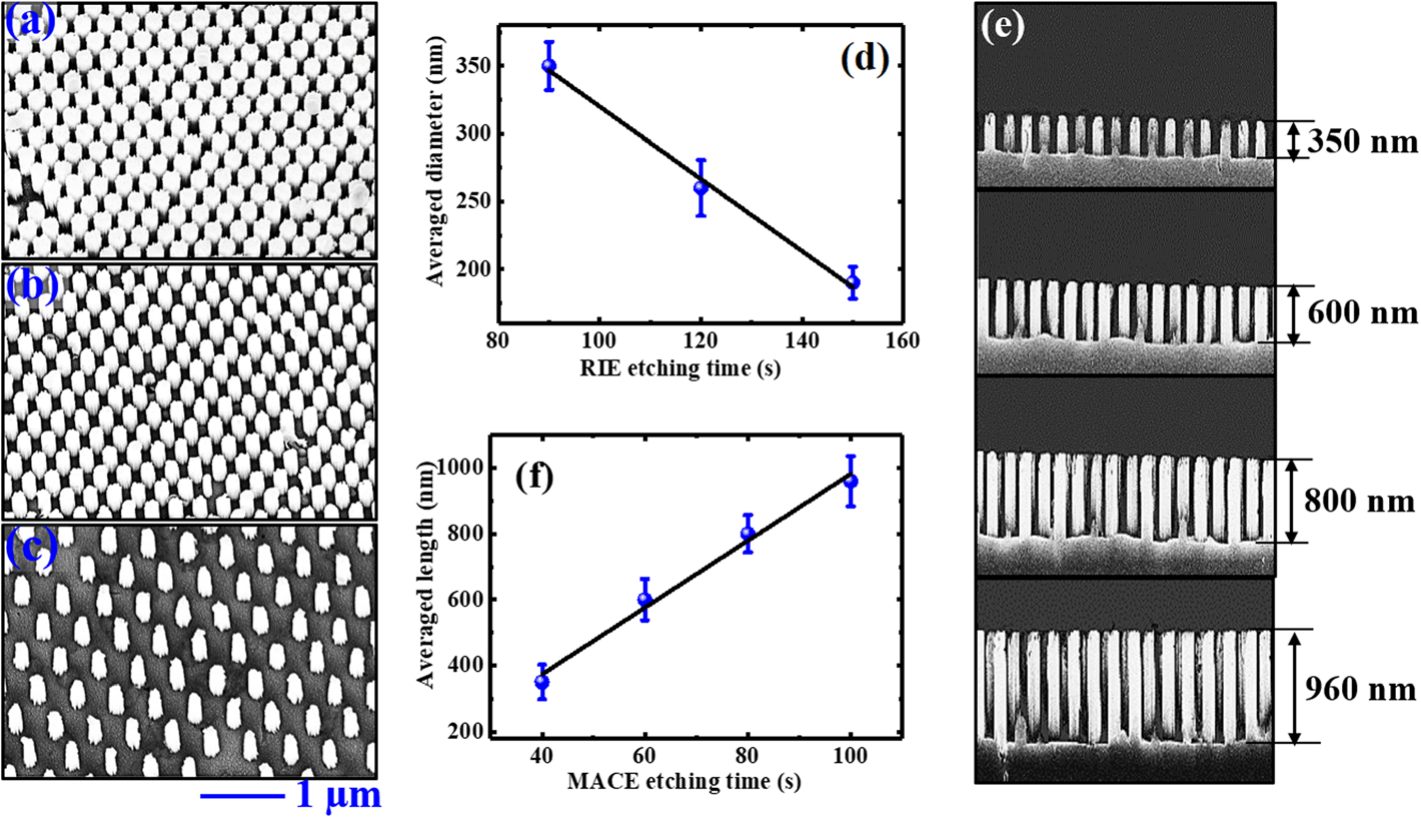
Top view SEM images of Si NWs with same length of 350 nm, but different diameters of **a** 350 nm; **b** 260 nm; and **c** 190 nm. The dependence of NWs’ diameter on the RIE etching time is plotted in **d**. **e** Cross-sectional SEM images of Si NWs with same diameter of 260 nm, but different lengths of 350, 600, 800, and 960 nm. **f** Presents the dependence of NWs’ length on the MACE time

The fabrication of large-area vertically aligned ordered Si nanowire arrays is illustrated in Fig. 1a. By changing the RIE time, the diameter of PS spheres can be reduced to desired values, and hence Si NWs with controllable diameters can be achieved. The SEM images of Si NWs obtained after 90, 120, and 150 s RIE etching are presented in Fig. 2 a, b, and c, respectively. It can be observed that the Si NWs are vertically aligned in a periodically hexagonal arrangement in a large area. These vertically aligned Si NWs have the same period of 490 nm and the same length of about 350 nm (same MACE time of 40 s). The diameters of the Si NWs in (a), (b), and (c) are around 350, 260 and 190 nm, respectively. The dependence of NWs’ diameter on the RIE etching time is plotted in Fig. 2d, showing a good linear correlation. On the other hand, the length of the nanowires could be adjusted by varying the time of MACE in HF and H_2_O_2_ mixed solution. The cross-sectional SEM images of Si NWs after 40, 60, 80, and 100 s MACE are shown in Fig. 2e. It can be seen that the NWs’ length increases from 350 to 960 nm by increasing the MACE time. Similarly, the Si NWs’ length exhibits a good linear dependence on the MACE time, as shown in Fig. 2f. These results indicate that ordered Si NWs with controllable diameters and lengths are successfully fabricated by the method of NSL combined with MACE.

**Fig. 3:**
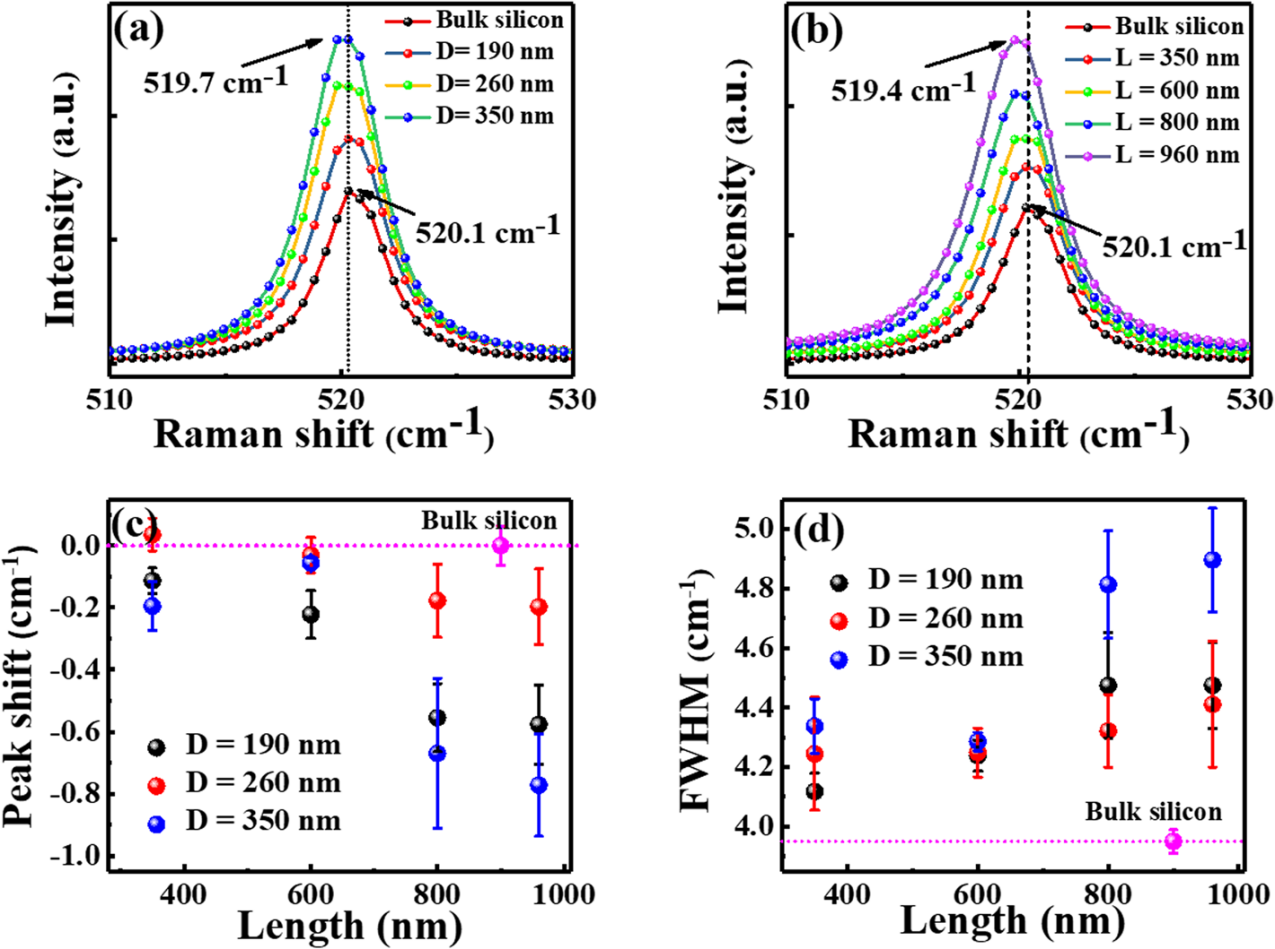
Typical Raman spectra of bulk Si and Si nanowires with **a** same length of 350 nm but different diameters and **b** same diameter of 190 nm but different lengths. **c** and **d** represent the redshift of peak position and the FWHM broadening as a function of nanowire length

From the SEM images, it can be also found that the Si NWs have a very rough surface after chemical etching, and their sidewall surface is especially rough. To check the microstructure of the surface layer as a function of diameter and length, Raman spectra were measured on both bulk Si and Si NWs with different diameters and different lengths. Each spectrum was normalized by using the maximum peak intensity at 520 cm^− 1^, and the results for different diameters and different lengths were shown in Fig. 3 a and b, respectively. The spectrum of bulk silicon shows a sharp peak locating at ~ 520.1 cm^− 1^. Both the redshift of peak position and broadening of peak width (termed with full-width at half-maximum, FWHM) can be observed on Si NWs, as plotted in Fig. 3 c and d, correspondingly. The peak redshift and broadening are rather small for nanowires with short lengths of 350 and 600 nm and become relatively obvious as the nanowire length increases to 800 nm and above. Such Raman peak redshift and broadening are sometimes attributed to the changes in dopant level or crystalline content. According to the previous literatures [5, 51], for the Si NWs fabricated by the same MACE method, the doping concentration could possess the same doping level as the starting wafer. Due to equipment limitations, we were unable to confirm this result. On the other hand, although these Si NWs have a rough surface, previous literatures found that the Si NWs fabricated by the MACE method could mainly keep their crystal structures for both n- and p-doping and both light and heavy doping [5, 51, 52]. Only a thin amorphous layer was observed on the wall surface of the NWs. Similar results were obtained on Si NWs with different diameters and lengths by different groups. As such, it could be assumed the Si NWs fabricated by the MACE method can keep their dopant level and crystalline content almost unchanged as their bulk counterpart, except the thin surface layer.

Therefore, the Raman peak shift and broadening are most probably induced by the nanowires’ damaged rough surface [53]. There are several papers reported the Raman results of Si NWs fabricated by the same MACE method. For example, Feser et al. found that the significant peak broadening at 520 cm^− 1^ increased with MACE time and attributed this peak broadening to the crystal disorder (e.g., clusters of point defects) induced by the etching process [23]. Lajvardi et al. found that Raman redshift increased with the MACE time, i. e. the TO Raman peak was located at 521.1 cm^− 1^ for bulk Si and shifted to 518.7 cm^− 1^ for 80-min etched samples, respectively [54]. They stated that the origin of the Raman shift may be due to the formation of Si nanocrystals on the wall of the nanowire. Lin et al. observed that the TO Raman peak shifted from 520 to 516 cm^− 1^ when the NWs’ length increases from 0 (bulk Si) to 2.73 μm, while the peak width was broadened from 3 to 9 cm^− 1^ with increasing NWs’ length from 0.19 to 2.73 μm [55]. They thought that the Raman peak broadening was dominated by the phonon-strain interaction and the Raman peak shift was proved to be proportional to the strain-induced surface lattice distortion. Since the strain induced by HF etching increased with the NWs’ length (more etching time), both the Raman peak redshift and broadening increased with the increasing NWs’ length. In our case, from the Raman spectra as shown in Fig. 3 a and b, we can find that the TO Raman peak shifts from 520 to 519.4 cm^− 1^ when the NWs’ length increases from 0 (bulk Si) to 960 nm, while the FWHM is broadened from 4.41 to 4.47 cm^− 1^ as NWs’ length increases from 350 to 960 nm. We prefer this length-dependent Raman peak shift and broadening are originated from the damaged surface (strain or disorder). However, due to the very small variation in both redshift and peak broadening for nanowires with different diameters and different lengths (< 1.0 cm^− 1^), the change of strain/disorder with size can be considered minimal. So the strain/disorder may modify the NWs’ conductance, but its influence on the size dependence of conductance is not concerned in the next sections.

### Conductive Property Measurements on Single Si NWs

**Fig. 4:**
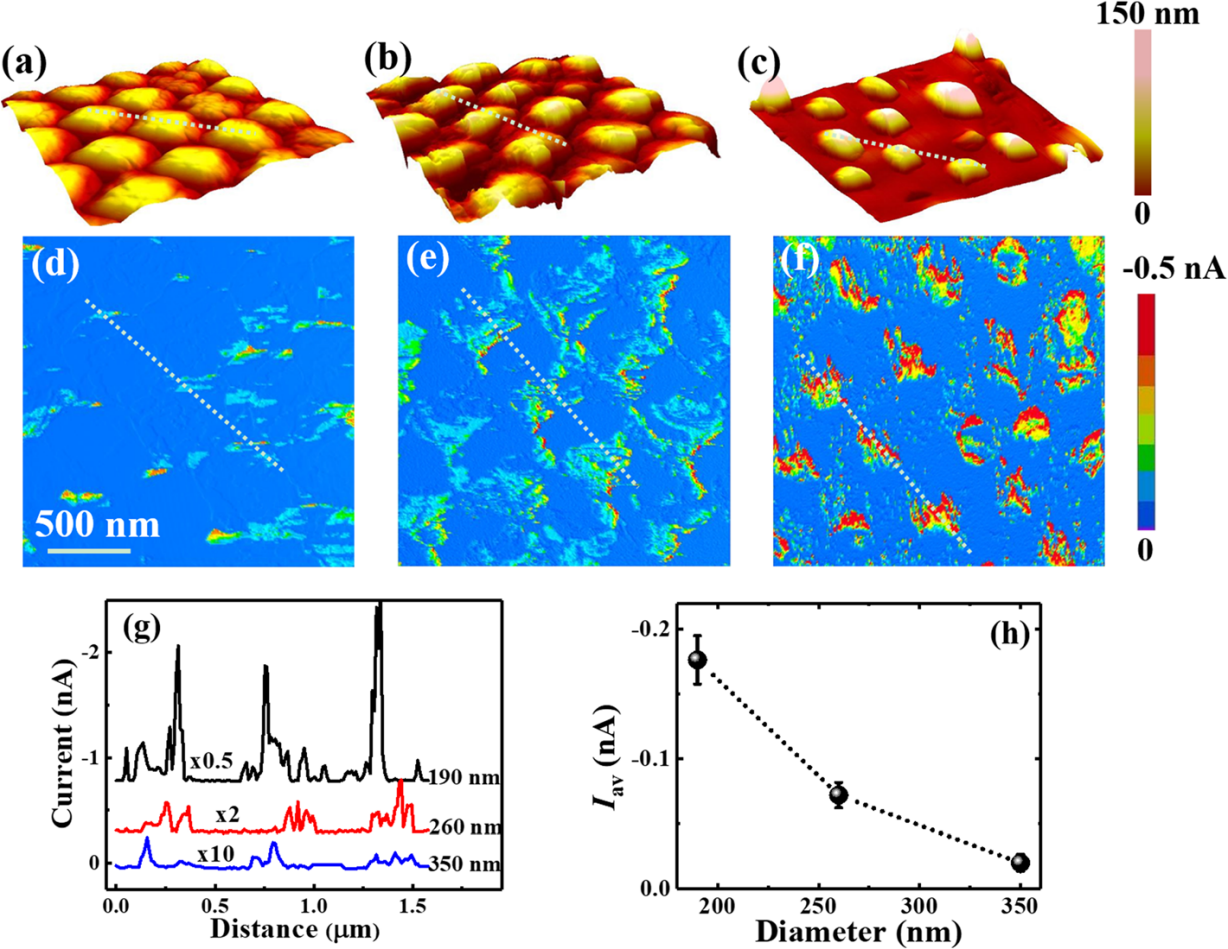
The topography images of Si NWs with the same length of 350 nm but different diameters of **a** 350 nm, **b** 260 nm, **c** 190 nm. Their corresponding current images obtained under the sample bias of − 1.5 V are given in **d**, **e** and **f**, respectively. Current profiles along the marked lines in **d**–**f** are plotted in **g**, and **h** presents the averaged current (*I*_av_) over the nanowires as a function of NWs’ diameter. Corresponding lines are added in the topography images of **a**–**c** and the profile curves in **g** are vertically shifted for guidance

The conductive properties of Si NWs are measured by CAFM on individual NWs with different diameters and lengths. Typical topography images of the vertically aligned Si NWs with the same length of 350 nm but different diameters of 350, 260, and 190 nm are shown in Fig. 4 a, b and c, respectively, while their corresponding current images obtained at the sample bias of − 1.5 V are presented in (d), (e), and (f). It should be noted, as the Cr/Pt-coated AFM tip is a wedge with a large angle, the Si NWs exhibit a larger diameter than their actual ones. Additionally, the current out of the nanowires could not be well detected as the tip may not be able to contact with the substrate, so only the currents measured on nanowires are taken into account. From the current images, it can be observed that most of the edges of Si nanowires exhibit a little better conductance than the center. This may be resulted by side contact between the AFM tip and the Si NW with a larger contact area. Additionally, due to the obvious roughness on the top surface, some regions at the center area may also show large current similar to the edge, resulting in no distinct ring-like current distribution. On the other hand, the conductance of Si NWs is obviously related to the nanowires’ diameter. It can be seen that both the conductive area ratio of nanowires and the absolute current values increase significantly as the diameter decreases from 350 to 190 nm. The results suggest that the Si NWs with smaller diameters are more conductive than those with larger ones. To get the diameter dependence more intuitively, the current profiles along the marked lines in Fig. 4 d to f are displayed in Fig. 4g. It clearly shows that the Si NWs with the diameter of 190 nm are much more conductive than those with the diameters of 260 nm and 350 nm. Such diameter dependence can also be obtained from the statistical histograms of current distributions on Si NWs with different diameters, as shown in Additional file 1: Figure S1(a), which exhibits the current distribution shifts to high values when the diameter decreases. The averaged currents (*I*_av_) of Si NWs are calculated by averaging the current over all the nanowires in the current images, which is plotted in Fig. 4h as a function of NWs’ diameter. The averaged current of Si NWs exhibits a dramatical nine times increase when the NWs’ diameter decreases from 350 to 190 nm. Similar current dependence on diameter has been achieved on single InAs nanowires as well as on single Si nanowire devices [35, 47].

**Fig. 5:**
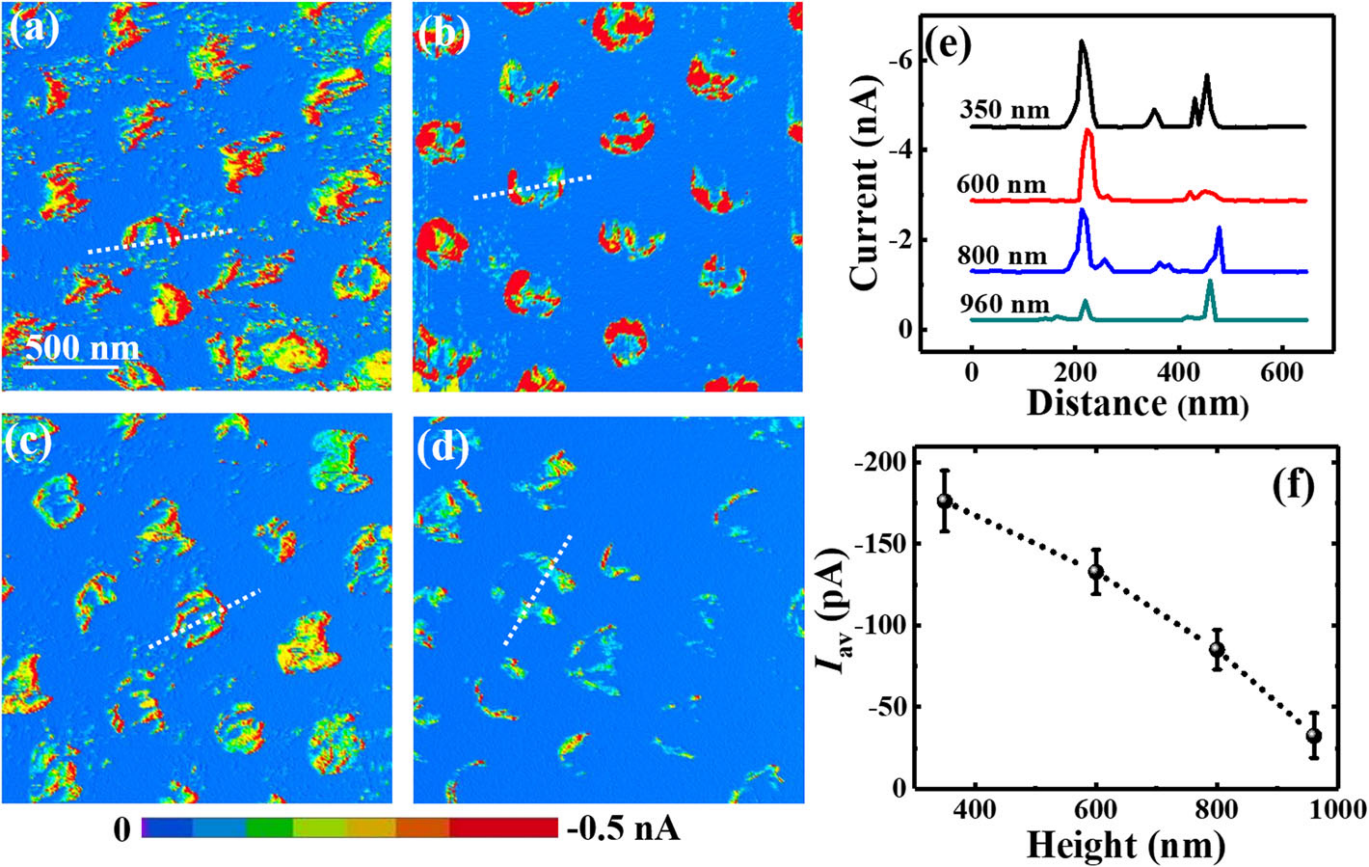
The current images of Si NWs under the sample bias of − 1.5 V with the same diameter of 190 nm but different lengths of **a** 350 nm, **b** 600 nm, **c** 800 nm, and **d** 960 nm, respectively. The current profiles along the marked lines in **a**–**d** are plotted in **e**, and **f** presents the averaged currents of the nanowires (*I*_av_) as a function of NWs’ length. The profile curves in **e** are vertically shifted for guidance

Typical current images of Si NWs with different lengths and the same diameter of 190 nm measured at the sample bias of − 1.5 V are presented in Fig. 5. Figure 5 a to d show the current images of Si NWs with the lengths of 350, 600, 800, and 960 nm, respectively. It can be seen that in these current images, the conductive areas decrease obviously with the increasing length, while the decrease of absolute current is not so obvious, especially for the NWs with the length of 350 nm and 600 nm. Maybe due to the existence of local irregular surface roughness, even larger current was observed at some spots in Fig. 5b. Nevertheless, the average current of Fig. 5b was much smaller than that of Fig.5a. Using the same analyses as above, the current profiles along the marked lines are presented in Fig. 5e, and the statistical histograms are shown in Additional file 1: Figure S1(b). Both of them clearly exhibit a significant current decrease with the increased NWs’ length. The averaged currents of the nanowires as a function of NWs’ lengths are plotted in Fig. 5f, and they are on the order of tens to hundreds pA which is much smaller than that shown in Fig. 5 a to e on the order of nA. It is because that the nanowires exhibit relatively large current only at a few conductive spots when most regions are nonconductive. From Fig. 5f, the averaged current exhibits more than three times decrease when the length increases from 300 to 960 nm, indicating the nanowires’ conductance decreasing with the increased length. The dependence of nanowire resistance on the length has previously been investigated by four-point resistance measurements on semiconductor nanowires, which suggested that under Ohmic contact the nanowire’ resistance increased with its length linearly with the slope of resistivity [56, 57]. In our case, from the plot of I ~ 1/L as given in Additional file 1: Figure S2, the dependence is remarkably nonlinear; hence, the resistivity could not be correctly obtained from the curve slope. It is worth note in CAFM measurements, the total measured resistance includes the contact resistance between Cr/Pt-coated tips and Si NW (*R*_tip/NW_), the resistance of Si NW (*R*_NW_) and the resistance of Si wafers (*R*_bulk_). Since the resistance measured by CAFM mainly comes from the localized surface area beneath the tip and it decreases quickly with the increased area along the current path, *R*_bulk_ is much smaller compared with *R*_NW_ and *R*_tip/NW_. On the other hand, due to the very small metal-semiconductor contact area between the tip and nanowire, the contact resistance *R*_tip/NW_ is much larger than the nanowire resistance *R*_NW_. The nonlinearity of current dependence on 1/L just indicates the presence of large contact resistance. Therefore, in the measurements carried out by CAFM, metal-semiconductor contact resistance should be emphatically considered, in which the Schottky barrier plays an important role.

**Fig. 6:**
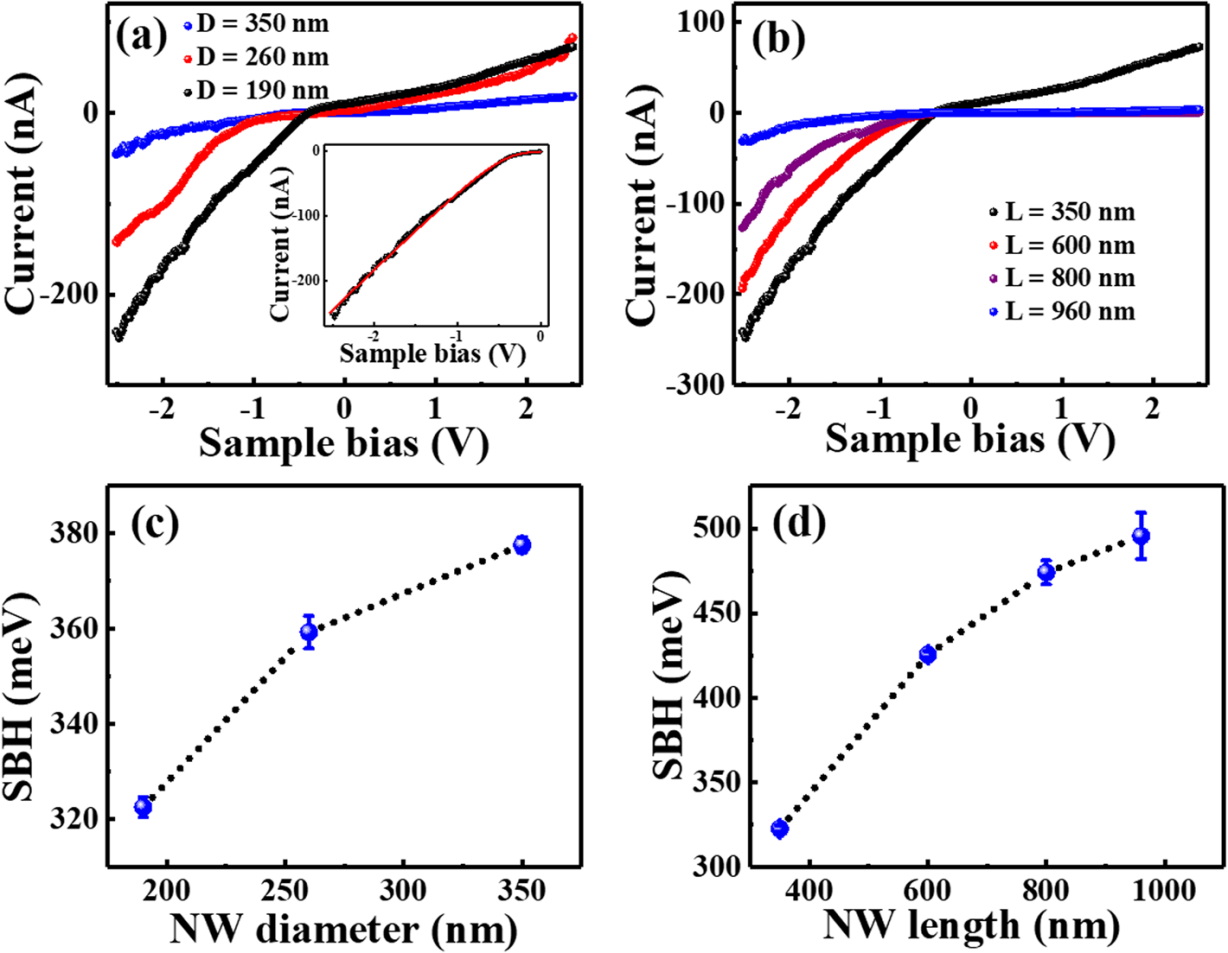
Typical I-V curves on the Si NWs with the same length of 350 nm but different diameters (**a**) and same diameter of 190 nm but different lengths (**b**). The inset in **a** shows a typical fitting result of Si NWs with the diameter of 190 nm and length of 350 nm. **c** and **d** represent the Schottky barrier heights obtained from the fitting results as a function of NWs’ diameter and length, respectively

To verify the above inference, current-voltage (I–V) curves were recorded on individual Si NWs to investigate the Schottky barrier at the metal tip/Si nanowire contact. Typical I–V curves on the Si NWs with the same length of 350 nm but different diameters are presented in Fig. 6a and those on the Si NWs with the same diameter of 190 nm but different lengths are displayed in Fig. 6b, respectively. All the I–V curves exhibit larger currents at negative sample voltage region, in accordance with the typical I–V curves with the Schottky contact between the metal tip and n-type semiconductor. As the I–V curves exhibit good metal-semiconductor characteristics, it indicates that the oxygen layer effect on conductance is not serious and thus assumed minimal in the following discussion. Meanwhile, the results show that smaller and shorter nanowires exhibit larger conductance than larger and longer ones, well consistent with the results obtained from current images. For quantitative analyses, a well-known thermionic emission model for a metal-semiconductor contact is adopted [58, 59]. In this model, the I–V characteristics of a Schottky contact to n-type semiconductor in the presence of series resistance can be approximated as [59]:

1 $$ I={I}_S\left[\exp \left(\frac{q\left(V-I{R}_S\right)}{\mathrm{n} kT}\right)-1\right], $$

where *n* is the ideal factor and *R*_S_ is the series resistance. *I*_S_ is the saturation current, which can be expressed by:

2 $$ {I}_S=\mathrm{AA}\ast {T}^2\exp \left(-\frac{\varphi_B}{kT}\right), $$

where A is the contact area, A* is Richardson’s constant, and ***φ***_B_ is the Schottky barrier height (SBH) between the metal tip and Si nanowire. Thus, SBH can be obtained with the formula:

3 $$ {\varphi}_B= kT\ln \left(\frac{\mathrm{AA}\ast {T}^2}{I_{\mathrm{S}}}\right), $$

The I–V curves in Fig. 6 a and b can be well fitted by Eq. (1), and a typical fitting line at the forward region is shown in the inset of Fig. 6a. To get the SBH values from the saturation current, the effective Richardson constant A* is assumed to be approximately equal to that of bulk silicon, i.e., 112 A cm^− 2^ K^− 2^ for n-type silicon [59]. The contact area is assumed to be 2 × 10^− 11^ cm^2^ by taking the Cr/Pt-coated tip radius as 25 nm. The SBH values are obtained to be about 322, 359 and 377 meV for the Si NWs with the same length of 350 nm and different diameters of 190, 260 and 350 nm, respectively. For Si NWs with the same diameter of 190 nm and different lengths of 350, 600, 800 and 960 nm, the SBH values are 322, 425, 473 and 495 meV, correspondingly. For comparison, typical I–V curve was measured on the same type of Si wafer, as shown in Additional file 1: Figure S3(a). It clearly shows that the conductance of Si wafer is much smaller than the produced NWs. Detectable current could only be measured at high bias voltages (− 4 ~ − 10 V). After fitting the I–V curves with the above thermionic emission model (Additional file 1: Figure S3(b)), SBH value of 0.60 eV was obtained for bulk Si. Obviously, all of the measured SBH values for Si NWs with different diameters and lengths are smaller than that of the bulk Si. Similar Schottky barrier lowering in nanowires has been reported by different groups on different types of nanowires, which was attributed to the carrier recombination in depletion region [46, 60], barrier inhomogeneity and Joule heating effect [48], or image potential lowering [47]. In our case, the barrier lowering can be also attributed to the large density of surface states induced image potential lowering and carrier charging in depletion region.

The dependence of SBH values on nanowires’ diameter and length is plotted in Fig. 6 c and d respectively, and it can be found that the SBH increases obviously with the increasing of both nanowires’ diameter and length. In addition, the same measurements were done on Si NWs with different diameters of 260 and 350 nm for all lengths, and the I–V curves are shown in Additional file 1: Figure S4 a and b, respectively. The obtained diameter-dependent SBH values from curve fitting for all lengths are listed in Table 1 and plotted in Additional file 1: Figure S5. The results show that the SBH values increase with increased diameter for all lengths, and also increase with increased length for all diameters. Therefore, the results obtained from the I–V curves analyses suggest that the Schottky barrier lowering is more significant for the nanowires with smaller diameters and shorter lengths. On the other hand, the ideal factor *n* and series resistances *R*s of Si NWs with different diameters and lengths can also be obtained from the fitting results, as listed in Table 1. The results show that *n* is much larger than 1 for all nanowires (2.8 ~ 9.4), indicating that the contact between the tip and nanowire is not ideal metal-semiconductor contact, probably due to the existence of oxide layer. On the other hand, the *R*s increases with increased diameter as well as increased length. For example, *R*_S_ increases from 6.1 to 21.6 MΩ as the diameter increases from 190 to 350 nm for the same length of 350 nm and increases from 6.1 to 32.3 MΩ for the length from 350 to 960 nm with the same diameter of 190 nm. The increase of *R*s with increased length is reasonable, while that increase with increased diameter is out of expectation. There is no good explanation for it at present, which may because that the series resistance is not simply the resistance of nanowire and the effective tip-nanowire contact area is not exactly equal to the nanowire’s sectional area. Nevertheless, the series resistances of Si NWs did be much smaller than the contact resistances, therefore the conductance of Si NWs should be dominated by the SBH determined contact resistance.

**Table 1:** Schottky barrier height (SBH), ideal factor (*n*) and series resistance (*R*s) obtained from the fitting results of I–V curves obtained on Si NWs with different diameters and lengths

Length & Diameter		SBH (meV)	*n*	*R*s (MΩ)
L=350 nm	D = 190 nm	323	5.7	6.1
	D = 260 nm	359	6.8	9.2
	D = 350 nm	378	9.4	21.6
L=600 nm	D = 190 nm	426	5.0	6.7
	D = 260 nm	438	7.1	31.9
	D = 350 nm	446	5.5	40.3
L=800 nm	D = 190 nm	474	4.1	11.0
	D = 260 nm	489	9.4	36.7
	D = 350 nm	542	7.7	40.4
L=960 nm	D = 190 nm	496	4.7	32.3
	D = 260 nm	544	6.2	181.3
	D = 350 nm	567	2.8	288.0

The origin of the size-dependent SBH is not very clear yet. The mechanism explanation for similar diameter dependence of SBH has been supposed in several literatures [45–48, 60]. For example, Leonard et al. interpreted this effect with the point of electron-hole recombination in depletion region [60]. As the recombination time decreased as the nanowire diameter was reduced, current density increased with decreasing nanowire diameter. Yoon et al. explained the diameter-dependent SBH using the presence of interface states [47]. Mao et al. attributed its origin to barrier inhomogeneity and Joule heating effect [48]. In our case, the Si NWs are made by the MACE method, so there inevitably exist an amount of defects on the surface and a reformed thin oxygen layer, resulting in a large density of surface states. Actually, from the enlarged SEM images and AFM observation, the top surface of Si NWs is very rough, further increasing the density of surface states. We think the presence of surface (or interface) states should be the main cause of the diameter dependence of SBH. According to previous literatures [47, 61, 62], the SBH lowering was interpreted by charged interface states. By adopting the cylindrical coaxial capacitor model used in reference [47], interface state–induced carrier transfer will form two opposite charged layers (metal and semiconductor contact surface) which generates an electric field opposite to the built-in electric field and lowers the barrier potential. As the surface state density increases with decreased nanowire diameter, smaller SBH is obtained on nanowires with a smaller diameter. Why the values of SBH related to the nanowire length is not clear yet. As the MACE time increased, the surface disorder or roughness increases correspondingly. Different changes in the surface microstructures may introduce different changes of SBH values, which need further investigations to work it out. Anyway, whatever the origin of size dependence of conductive properties, the size-dependent SBH lowering could result in higher conductance, which should be beneficial for practical applications.

### EFM Measurements on Single Si NWs

To further verify the size-dependent SBH results of Si NWs obtained by CAFM, the EFM measurements were performed on the same samples and the EFM phase shift was measured as a function of applied DC bias. In previous literatures [63, 64], the relation between phase shift and electrostatic force has already been established, where the tip-sample system is roughly treated as a plane capacitor. When a bias is applied between the tip and the sample, the capacitive electrostatic force gradient would cause a phase shift. At a lifted height where the Van de Waals force can be ignored, the electrostatic force acted on the tip can be expressed as [63]:

4 $$ F=\frac{1}{2}\frac{\partial C}{\partial z}{\left({V}_{EFM}-{V}_{CPD}\right)}^2, $$

where *C*, *V*_EFM_, and *V*_CPD_ are the capacitance, applied DC voltage and contact potential difference (CPD) between the sample and tip, respectively. *V*_CPD_ can be written as (***φ***_sample_ − ***φ***_tip_)/e when the bias voltage *V*_EFM_ was applied to the sample in our experiments. The phase shift detected by EFM is proportional to the gradient of the electrostatic force, which can be written as:

5 $$ \varDelta \varPhi =-\frac{Q}{k}\frac{\partial F}{\partial z}=-\frac{Q}{k}\left[\frac{1}{2}\frac{\partial^2C}{\partial {z}^2}{\left({V}_{EFM}-{V}_{CPD}\right)}^2\right], $$

where *Q* is the quality factor, *k* is the spring constant of the probe and *z* is the distance between tip and top of Si NW.

**Fig. 7:**
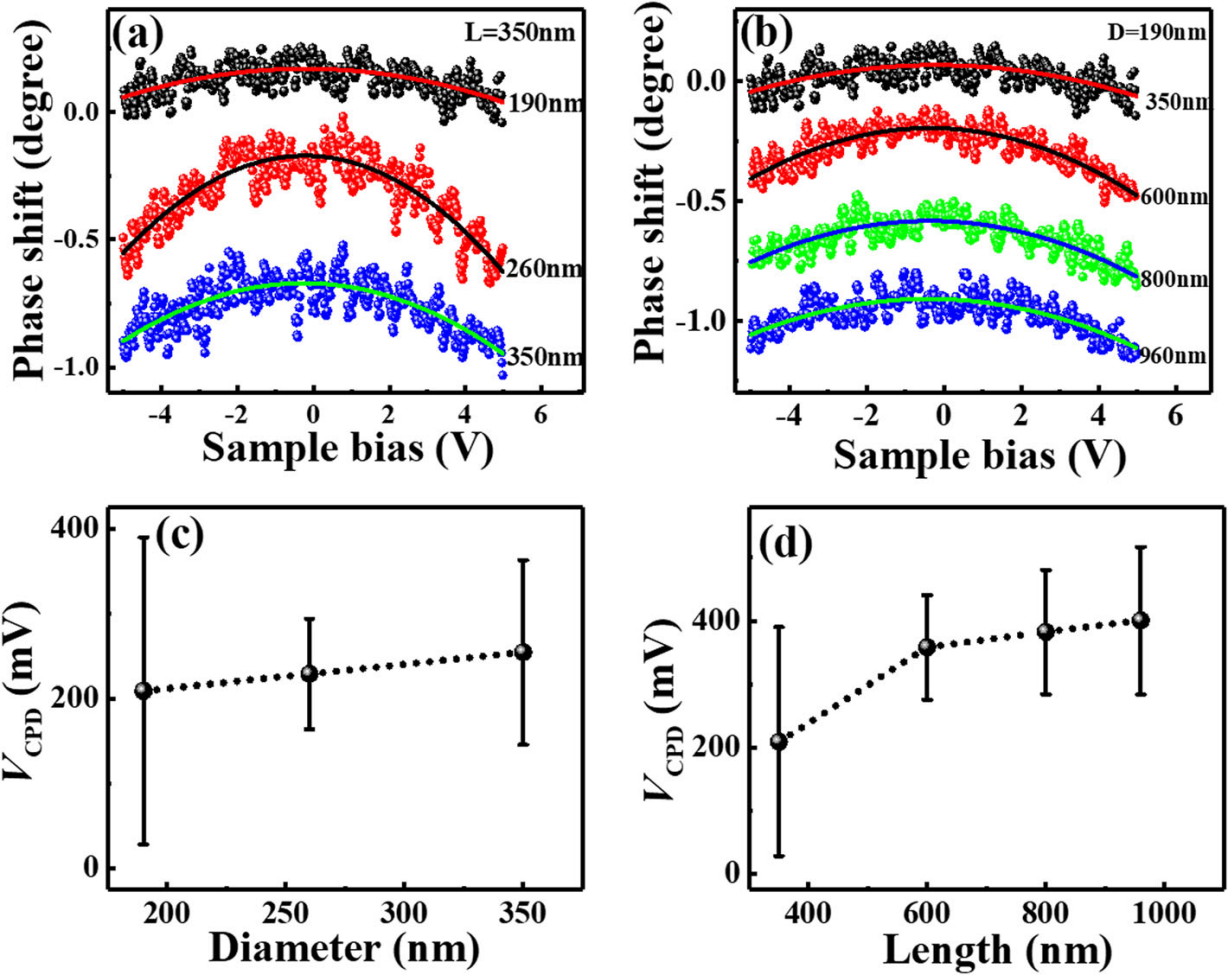
∆*Φ* ~ *V* curves measured by EFM on individual Si NWs with **a** different diameters of 190, 260, and 350 nm (length = 350 nm) and **b** different lengths of 350, 600, 800, and 960 nm (diameter = 190 nm). **c** and **d** present the *V*_CPD_ values obtained by curve fitting as a function of NWs’ diameter and length. The curves in **a** and **b** are vertically shifted for guidance

From Eq. (5), it can be seen that the EFM phase shift should be equal to zero at *V*_EFM_ = *V*_CPD_. Therefore, *V*_CPD_ can be achieved from the EFM measurements. The Δ*Φ*~*V*_EFM_ curves measured at a lift height of 100 nm on the individual Si NWs with different diameters and lengths are shown in Fig. 7 a and b as the scattered dots, respectively. By using Eq. (5), the *ΔΦ*~*V*_EFM_ curves can be fitted well, shown as the solid lines in Fig. 7 a and b. From the fitting parameters, the values of *V*_CPD_ can be obtained, as presented in Fig. 7 c and d as a function of diameter and length respectively. The results show that the CPD values increase with increased diameter and increased length. Similar CPD results have been reported in a previous work performed by Kelvin probe force microscopy on ZnO NWs, in which the absolute value of CPD between ZnO nanowire and Pt/Ir tip also increased with increased diameter [65]. As diagrammed in Additional file 1: Figure S6, the value of SBH roughly equals to *qV*_CPD_ plus *E*_n_ (= *E*_C_ − *E*_F_). As *E*_n_ is a constant for all Si NWs made from the same material, the size dependence of *V*_CPD_ well represents the size dependence of SBH. Therefore, from the EFM results, it can be suggested that, the SBH values increase with the increasing of NWs’ diameter and length, well consistent with the results achieved by CAFM measurements. Similarly, the EFM measurements are performed on all series of Si NWs, and the diameter-dependent *V*_*CPD*_ values at different lengths are shown in Additional file 1: Figure S7(a) and (b) respectively, which exhibit same diameter dependence as that obtained by CAFM.

## Conclusion

In summary, by a simple, low-cost method without involving any intricated procedures, Si NWs arrays with controllable diameters and lengths are prepared. Both the diameter and length of SiNWs can be well controlled by adjusting the etching time. The conductive properties include the current map and I–V curves are directly measured on individual Si NWs without complex nanofabrication procedure by the means of CAFM. Size-dependent conductance of Si NWs can be obtained from both the current images and I–V curves. Our results demonstrate that the Si NWs with a smaller diameter and shorter length exhibit better conductance. It can be attributed to the size dependence of SBH, which increased from 322 to 377 meV with the diameter increasing from 190 to 350 nm for the same length of 350 nm. Correspondingly, the SBH values increased from 322 to 495 meV as the length varies from 350 to 960 nm for the same diameter of 190 nm. The same size-dependent SBH can also be obtained from the EFM measurements. Such SBH lowering is interpreted by charged interface states. Therefore, our study not only reveals the size-dependent properties of Si NWs but also suggests that CAFM can act as an effective means to explore the size (or other parameters) dependence of conductive properties on individual nanostructures.

## Supplementary information

**Additional file 1: Figure S1.** Histograms of the current distribution of Si NWs obtained from the current images as shown in Figures 3 and 4. (a) and (b) presents the statistical current distributions of Si NWs with different diameters (same length of 350 nm) and different lengths (same diameter of 190 nm), respectively. The current distribution shifts to right with decreased diameter and length. **Figure S2.** Averaged current as a function of 1/Length. **Figure S3.** (a) Typical I-V curve measured on the same type of Si wafer; (b) Fitting result of the I-V curve in (a) with equation (1). **Figure S4.** Typical I-V curves on the Si NWs with different lengths and the same diameter of 260 nm (a) and 350 nm (b), respectively. The curves exhibit similar length dependence as that obtained from Fig. 6b. **Figure S5.** Diameter and length dependent Schottky barrier heights obtained from the fitting results of I-V curves on Si NWs with different lengths and different diameters are shown in (a) and (b), respectively. **Figure S6.** Energy band diagram of the contact interface between metallic tip and Si NW. *V*_*CPD*_ is the contact potential difference and the value of SBH roughly equals to the sum of *qV*_*CPD*_ and *E*_*n*_. **Figure S7.** Diameter and length dependent contact potential difference values obtained by EFM curve fitting are given in (a) and (b), respectively.

## Data Availability

The datasets used for supporting the conclusion are included in the article and the supporting file.

## Abbreviations

CAFM: Conductive atomic force microscopy
CPD: Contact potential difference
EFM: Electrostatic force microscopy
MACE: Metal-assisted chemical etching
NSL: Nanosphere lithography
PS: Polystyrene spheres
RIE: Reactive ion etching
SBH: Schottky barrier height
SEM: Scanning electron microscopy
Si NWs: Si nanowires
SPM: Scanning probe microscopy
