# Supplementary material for: Investigating Size-Dependent Conductive Properties on Individual Si Nanowires
Source: Nanoscale Res Lett. 2020 Mar 2;15:52. doi: 10.1186/s11671-020-3277-3 (PMC7052096; doi:10.1186/s11671-020-3277-3)
Supplement: Supplementary file 1 — Additional file 1: Figure S1. Histograms of the current distribution of Si NWs obtained from the current images as shown in Figures 3 and 4. (a) and (b) presents the statistical current distributions of Si NWs with different diameters (same length of 350 nm) and different lengths (same diameter of 190 nm), respectively. The current distribution shifts to right with decreased diameter and length. Figure S2. Averaged current as a function of 1/Length. Figure S3. (a) Typical I-V curve measured on the same type of Si wafer; (b) Fitting result of the I-V curve in (a) with equation (1). Figure S4. Typical I-V curves on the Si NWs with different lengths and the same diameter of 260 nm (a) and 350 nm (b), respectively. The curves exhibit similar length dependence as that obtained from Fig. 6b. Figure S5. Diameter and length dependent Schottky barrier heights obtained from the fitting results of I-V curves on Si NWs with different lengths and different diameters are shown in (a) and (b), respectively. Figure S6. Energy band diagram of the contact interface between metallic tip and Si NW. VCPD is the contact potential difference and the value of SBH roughly equals to the sum of qVCPD and En. Figure S7. Diameter and length dependent contact potential difference values obtained by EFM curve fitting are given in (a) and (b), respectively. [file 11671_2020_3277_MOESM1_ESM.pdf]

Investigating size-dependent conductive properties on individual Si nanowires

X. F. Hu, S. J. Li, J. Wang, Z. M. Jiang, and X. J. Yang[*]

State Key Laboratory of Surface Physics, Fudan University, Shanghai 200433, China

[*] Corresponding author. E-mail address: xjyang@fudan.edu.cn.

1. Statistical current distributions of Si NWs with different diameters and lengths

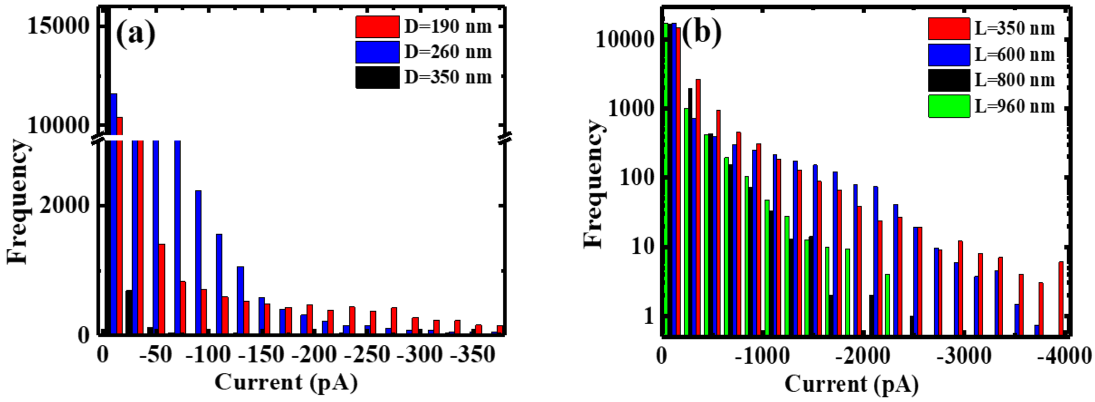

Figure S1. Histograms of the current distribution of Si NWs obtained from the current images as shown in Figures 3 and 4. (a) and (b) presents the statistical current distributions of Si NWs with different diameters (same length of 350 nm) and different lengths (same diameter of 190 nm), respectively. The current distribution shifts to right with decreased diameter and length.

2. The dependence of averaged current on NWs' length

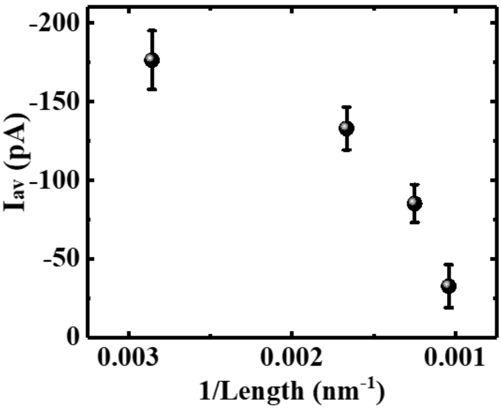

Figure S2. Averaged current as a function of 1/Length.

3. I-V characteristics of bulk Si

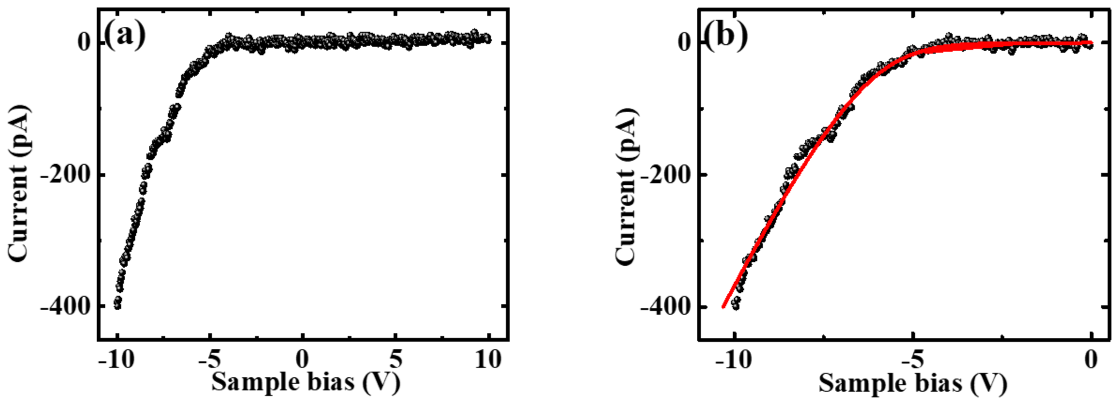

Figure S3. (a) Typical I-V curve measured on the same type of Si wafer; (b) Fitting result of the I-V curve in (a) with equation (1).

4. Length-dependent I-V curves of Si NWs with different diameters

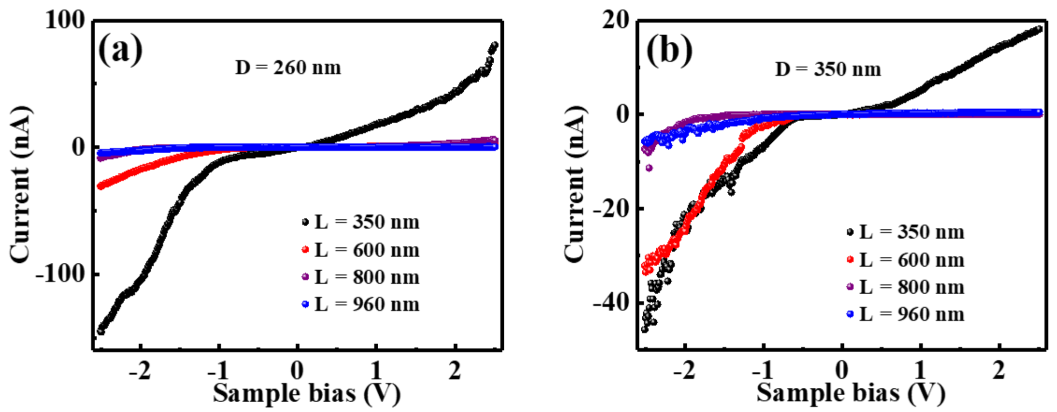

Figure S4. Typical I-V curves on the Si NWs with different lengths and the same diameter of 260 nm (a) and 350 nm (b), respectively. The curves exhibit similar length dependence as that obtained from Figure 6(b).

5. Diameter and length dependent Schottky barrier heights obtained on different series of Si NWs

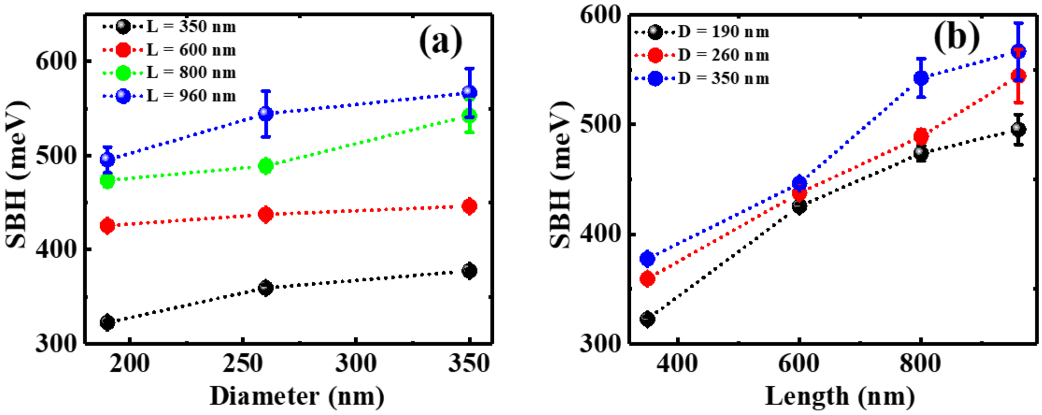

Figure S5. Diameter and length dependent Schottky barrier heights obtained from the fitting results of I-V curves on Si NWs with different lengths and different diameters are shown in (a) and (b), respectively.

6. Schematic energy band diagram of the tip-sample contact

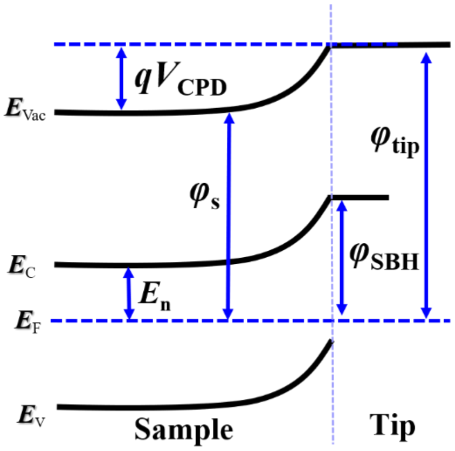

Figure S6. Energy band diagram of the contact interface between metallic tip and Si NW. V_{CPD} is the contact potential difference and the value of SBH roughly equals to the sum of qV_{CPD} and E_n.

7. Diameter and length dependent contact potential difference values obtained on different series of Si NWs.

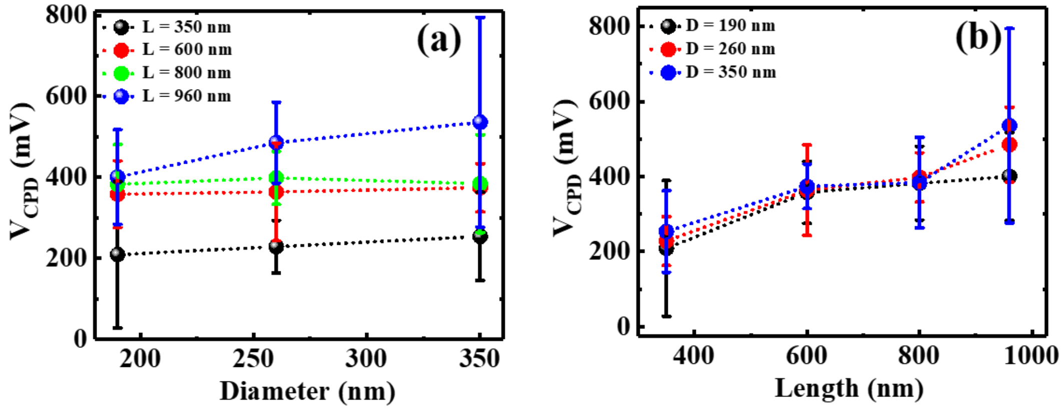

Figure S7. Diameter and length dependent contact potential difference values obtained by EFM curve fitting are given in (a) and (b), respectively.
